# Carrier frequency of autosomal recessive monogenic disorders in the peruvian population

**DOI:** 10.3389/fgene.2026.1876411

**Published:** 2026-07-23

**Authors:** Hugo Hernán Abarca Barriga, Flor Del Milagro Vásquez-Sotomayor, Renzo Punil Luciano

**Affiliations:** 1 Instituto de Investigaciones en Ciencias Biomédicas, Universidad Ricardo Palma, Santiago de Surco, Lima, Peru; 2 Servicio de Genética & Errores Innatos del Metabolismo, Instituto Nacional de Salud del Niño, Breña, Lima, Peru

**Keywords:** exome sequencing, genetic carrier screening, heterozygote, Latin America, population

## Abstract

**Objective:**

Autosomal recessive diseases represent an important group of genetic disorders whose frequency may be reduced through population screening strategies or premarital diagnosis, particularly in low- and middle-income countries, where some of these conditions have personalized treatment options that are often not covered by healthcare systems. The aim of this study was to determine the frequency of carriers of autosomal recessive monogenic disorders among patients who underwent exome sequencing at a national pediatric referral hospital in Peru.

**Materials and Methods:**

An observational study was conducted in which carrier frequency was calculated based on pathogenic or likely pathogenic variants identified through exome sequencing. Additionally, assumptions from the Hardy-Weinberg equilibrium were applied to estimate the expected frequency of affected individuals in the population. Exploratory analyses were performed to estimate the odds ratio to evaluate the possible association between the geographic origin of patients or their grandparents and the presence of recessive variants.

**Results:**

The sample included 178 patients who underwent exome sequencing. In the analyzed cohort, approximately one pathogenic or likely pathogenic heterozygous variant per individual was identified, and at least 63.5% of participants were carriers of at least one genetic condition.

**Conclusion:**

Carrier frequencies for genes such as *GJB2*, *PAH*, and *ABCA4* were slightly lower than those previously reported. Similarly, the estimated prevalence of potentially affected individuals showed variations compared with previous studies across different disease groups. These differences may be explained by demographic and evolutionary factors that influence population genetic structure.

## 1 Introduction

Rare diseases have an estimated prevalence of 2%–6% in the general population, of which up to 80% have a genetic origin (Jobanputra et al., 2024). Autosomal recessive or X-linked diseases account for approximately 35% of monogenic disorders, with a combined population prevalence ranging from 0.1% to 0.3% (Yang et al., 2014; Hanany et al., 2020; Fridman et al., 2021; Quaio et al., 2021; Quaio et al., 2022; Choi et al., 2024). The frequency of pathogenic or likely pathogenic variants associated with autosomal recessive or X-linked diseases varies across populations. Studies based on next-generation sequencing have estimated that between 20% and 36% of the general population may carry at least one variant associated with a recessive disease (Hanany et al., 2020; Schmitz et al., 2022; Choi et al., 2024; Hotakainen et al., 2025).

Among potentially preventable conditions are those in which a family history exists, whether inherited in a dominant or recessive manner. However, in most patients evaluated with recessive diseases, recurrence risk cannot be determined. In this context, preconception diagnosis allows identification of carrier status and assessment of reproductive risk before conception. This approach is more effective than prenatal screening because it allows identification of severe diseases and may influence family planning decisions (Capalbo et al., 2021; Sagaser et al., 2023; Peterlin and Peterlin, 2025).

Peru has a highly heterogeneous population characterized by a complex demographic and partly due to diverse ethnic backgrounds, including Native American, European, African, and Asian ancestral contributions. The relative contribution of these ancestral components varies across geographic regions, particularly between the coast, Andean, and Amazon (Borda et al., 2026). Such population structure may influence the distribution of pathogenic variants and carrier frequencies of monogenic disorders, underscoring the importance of generating population-specific genomic data. Consequently, estimates derived from European, North American, or Asian populations may not accurately represent the genetic architecture of Latin American populations. Despite the growing implementation of next-generation sequencing technologies worldwide, data on carrier frequencies in Peru remain unavailable. Until recently, exome sequencing was not routinely available within the Peruvian public healthcare system, limiting the generation of local genomic data and population-specific estimates of carrier frequencies. Although international commercial laboratories have offered exome sequencing services for several years, access has remained restricted because of economic and logistical barriers.

The implementation of genomic technologies in low- and middle-income countries faces several challenges, including limited infrastructure, restricted access to sequencing platforms, shortages of trained personnel in genomic medicine and bioinformatics, and insufficient resources for large-scale genomic research. These limitations contribute to disparities in diagnostic capabilities and hinder the generation of local genomic evidence (Fajardo and Abarca-Barriga, 2021). Expanding access to exome sequencing in countries such as Peru may not only improve the diagnosis of genetic disorders but also facilitate the development of carrier screening programs, genetic epidemiology studies, and precision medicine initiatives tailored to local populations.

In Peru, there are no previous studies that determine carrier frequency using exome sequencing. In Brazil, however, the carrier frequency of at least one pathogenic or likely pathogenic variant associated with autosomal recessive diseases has been estimated at 71.9%, suggesting that individuals affected by an autosomal recessive condition represent approximately 0.26% of the population (Quaio et al., 2021).

The objective of this study was to determine the frequency of carriers of autosomal recessive diseases in patients who underwent exome sequencing at the national pediatric referral hospital in Peru.

## 2 Materials and Methods

### 2.1 Study design

An observational, cross-sectional study was conducted in a clinical cohort of pediatric patients with suspected genetic disorders who underwent exome sequencing (ES) at the national pediatric referral hospital in Peru.

### 2.2 Setting

The study was carried out at the Servicio de Genética y Errores Innatos del Metabolismo (SGEIM) of the Instituto Nacional de Salud del Niño (INSN) in Breña-Lima. This is the National Pediatric Referral Hospital in Peru and the only one offering exome sequencing in the country. Consequently, patients were referred from multiple regions nationwide.

### 2.3 Participants

The study sample consisted of a convenience clinical cohort of 178 patients evaluated for suspected genetic disorders who underwent ES between 2020 and 2022.

### 2.4 Inclusion criteria

Patients who underwent detailed clinical evaluation an ES at INSN-Breña between 2020 and 2022, and parental informed consent authorizing the use of data for research purposes.

### 2.5 Exclusion criteria

Patients with inadequate sequencing quality metrics.

### 2.6 Variables

Demographic variables analyzed were sex, age (measured at the time of sample collection), and place of origin (classified by department, natural region, and poverty level). The primary variable of interest was the presence of pathogenic or likely pathogenic single nucleotide variants (SNV) in genes associated with autosomal recessive diseases, which were classified following ACMG guidelines. Variants considered causative of the patients’ presenting phenotypes were excluded from this analysis. The study included only heterozygous pathogenic or likely pathogenic variants in genes associated with autosomal recessive disorders that were unrelated to the clinical indication for ES, which were defined as carrier findings. All variants were classified according to ACMG/AMP (guidelines 2015 standards and subsequent updates). In addition, a variant was considered novel if it was absent from gnomAD and had not been previously reported in ClinVar or indexed scientific literature.

### 2.7 Data sources

Data were obtained from ES studies performed at the molecular laboratory of SGEIM and which files were stored *in situ*. Briefly, genomic DNA was isolated from whole blood using the gSYNC™ DNA Extraction Kit (Geneaid Biotech Ltd., New Taipei City, Taiwan). DNA concentrations were determined using Qubit Assays (Thermo Fisher Scientific, United States). The Ion AmpliSeq Exome RDY Kit (Thermo Fisher Scientific, United States), which targets 19,072 genes, was used for exome enrichment and library construction. The sample was ligated with an adapter and barcode using the Ion Xpress kit, and DNA purification was performed with Agencourt Ampure XP beads (Beckman Coulter, Indianapolis, IN, United States). The Ion 540™ Kit-Chef was used for templating and chip loading for sequencing. Exome sequencing was performed using a 400 bases read length and a total of 520 flows on an Ion GeneStudio S5 sequencer (Life Technologies). Reads were aligned to the hg19 reference genome. Tertiary analysis was performed in 2020–2022 and FASTQ files were stored at SGEIM.

### 2.8 Analysis procedure

Bioinformatic reanalysis was performed between July and September 2025 using Bitgenia® software. Quality control, read mapping, variant calling, and variant annotation were performed internally by the Bitgenia® cloud-based platform; the specific tools and versions used are proprietary and not publicly disclosed. Quality filters included read depth equal to or greater than 20x and allele fraction greater than 0.3. Homopolymeric regions were excluded due to limitations inherent to the Ion Torrent platform, and therefore, reanalysis of insertion or deletion variants causing frameshift mutations in these regions was not performed. For each case, candidate variants were identified through three parallel filter groups: (1) variants previously classified as pathogenic or likely pathogenic at least once in ClinVar; (2) nonsense and frameshift variants; and (3) missense variants with a Bayesian score greater than 0.8 and/or classified as pathogenic or likely pathogenic by the Bitgenia® platform. All candidate variants were then manually reviewed using the Integrative Genomics Viewer (IGV) embedded within the Bitgenia® platform and subsequently classified following the American College of Medical Genetics and Genomics guidelines v3.2 (ACMG). Final pathogenicity classification was confirmed using Franklin v81 and VarSome v13.5. Variants classified as benign or likely benign by at least one tool were excluded. Variants of unknown significance (VUS) were not included in the analysis because their clinical relevance remains unresolved and their inclusion could lead to overestimation of carrier frequencies and disease prevalence.

### 2.9 Bias

Inclusion and exclusion criteria were standardized. Variant review was independently conducted by 2 trained professionals and subsequently evaluated by a third reviewer. Standardized international databases such as ClinVar and OMIM were used. Because the study included patients referred for exome sequencing based on clinical suspicion of genetic disease, selection bias is inherent to the study design. Therefore, the results should be interpreted as representative of a clinical referral cohort rather than of the general Peruvian population.

### 2.10 Sample size

The sample size corresponded to a convenience cohort including all patients who met inclusion criteria during the study period.

### 2.11 Statistical methods

Descriptive statistics were used to determine the absolute and relative frequency of variants. Carrier frequency (c) was calculated as the proportion of heterozygous carriers (n) over the total sample size (N). Because the study population consisted of a clinically selected referral cohort rather than a random population sample, Hardy–Weinberg equilibrium-based calculations were performed for exploratory and descriptive purposes only. Consequently, the resulting estimates should not be interpreted as population-based prevalence estimates for Peru. Assuming Hardy–Weinberg equilibrium (HWE), the pathogenic allele frequency (q) was estimated by solving the equation c = 2q (1−q), where c represents the observed carrier frequency. If homozygous individuals had been detected, they would have been accounted for in the total allele frequency calculation, and the expected frequency of affected individuals was then calculated as q^2^. All mathematical equations and HWE estimations were implemented and calculated using Microsoft Excel (v.16.109.2). To evaluate the homogeneity of carrier distribution, a chi-square goodness-of-fit test was applied, comparing proportions according to department and natural region against expected distributions based on data from the *Instituto Nacional de Estadística e Informática* (*INEI*). Additionally, the unadjusted bivariate association between carrier status and geographic departments or natural regions was explored using the Chi-square test (or Fisher’s exact test, where appropriate). The estimated Peruvian population used for calculations was obtained from INEI, considering a total of 34,350,244 individuals. Carrier frequencies were calculated from the study sample and, assuming Hardy–Weinberg equilibrium, the expected frequency of affected individuals was estimated. The expected numbers of carriers and affected individuals in Peru were subsequently projected using the national population estimates provided by INEI. These projections should be interpreted as theoretical estimates derived from this clinical cohort and not as population-based estimates for Peru. A value of p < 0.05 was considered statistically significant, with a 95% confidence level. Stata version 18 was used for data analysis.

### 2.12 Ethical considerations

The study was approved by the Research Ethics Committee of INSN-Breña (Official letter No. 164-2025-CIEI-INSN) and conducted in accordance with the Declaration of Helsinki and national regulations. All participants had informed consent signed by parents or legal guardians, including authorization for secondary data use for research purposes. Confidentiality was ensured through coded identifiers and secure data storage systems.

## 3 Results

The mean number of pathogenic or likely pathogenic variants per individual related to autosomal recessive diseases was 0.96 (SD = 0.95). A total of 63.5% (n = 113) of patients had at least one variant related to a recessive disease. Additionally, 7.3% (n = 13) of evaluated individuals had three or more variants, with some patients presenting up to five variants. A total of 170 variants were identified, most of which were classified as likely pathogenic (n = 158, 92.9%), while 12 (7.1%) were categorized as pathogenic. Regarding variant type, nonsense variants were the most frequent (n = 77, 45.3%), followed by missense variants (n = 66, 38.8%) and splice-site alterations (n = 27, 15.9%) (Supplementary Material S1).

**FIGURE 1:**
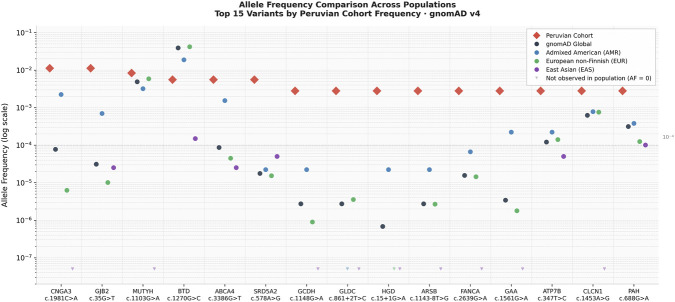
Comparison of allele frequencies of the most frequent pathogenic and likely pathogenic variants identified in the Peruvian cohort and reference populations from gnomAD. Allele frequencies are shown for the 15 variants with the highest frequencies in the study cohort and are compared with frequencies reported in the gnomAD Global, Admixed American (AMR), Non-Finnish European (NFE), and East Asian (EAS) populations. Variants not observed in each population are indicated as AF = 0. The y-axis is displayed on a logarithmic scale to facilitate comparison across variants with different frequency ranges.

Among the unique variants detected, 28 were considered novel because they were absent from gnomAD, ClinVar, and indexed scientific publications. In addition, 61 variants were not observed in the Admixed American (AMR) population of gnomAD. Comparison with gnomAD reference populations showed that only seven variants had allele frequencies lower than those reported in the gnomAD Global dataset, whereas the remaining variants exhibited higher observed allele frequencies within the study cohort (Supplementary Material S1). The 15 most frequent variants identified in the cohort showed heterogeneous allele frequency distributions across gnomAD populations, with several variants displaying higher frequencies in the Peruvian cohort than in the AMR, European, East Asian, or global reference populations (Figure 1).

The geographic origin of patients and their grandparents was predominantly from the coastal region, exceeding what would be expected according to the distribution of the Peruvian population (Table 1).

**TABLE 1:** General characteristics of Peruvian patients who underwent exome sequencing.

Characteristic	Mean (SD)	Median (IQR)	P value
Age^a^	9.26 (4.87)	8 (8)	​
Number of variants^ζ^	0.96 (0.95)	1 (1)	​
Frequency of variants per patient	**n**	**%**	​
0	65	36.5	​
1	71	39.9	​
2	29	16.3	​
3	11	6.2	​
4	1	0.6	​
5	1	0.6	​
Sex	​	​	​
Male	104	58.4	0.025^b^
Female	74	41.6	
Origin	​	​	​
Coast	151	84.8	<0.001^b^
Andean	17	9.6	
Amazon	10	5.6	
Maternal grandmother origin	​	​	​
Coast	47	45.6	0.008^b^
Andean	41	39.8	
Amazon	15	14.6	
Maternal grandfather origin	​	​	​
Coast	47	45.2	0.002^b^
Andean	44	42.3	
Amazon	13	12.5	
Paternal grandmother origin	​	​	​
Coast	47	47.9	<0.001^b^
Andean	38	38.8	
Amazon	13	13.3	
Paternal grandfather origin	​	​	​
Coast	49	49.5	<0.001^b^
Andean	37	37.4	
Amazon	13	13.1	

^a^All measures of central tendency and dispersion are presented, although the variable did not show a normal distribution.

^b^ Chi-square goodness-of-fit test comparing the observed sample distribution with the expected population distribution derived from National Institute of Statistics and Informatics (INEI) data.

The estimated number of individuals affected by an autosomal recessive disease in Peru exceeds 79,384 (Table 2). The most frequent conditions are associated with prelingual sensorineural hearing loss caused by variants in *GJB2* (Table 2). Additionally, diseases affecting the retina and those associated with male infertility were also frequently observed (Table 2). In addition to heterozygous carrier findings, eight pathogenic or likely pathogenic variants were identified in the homozygous state (Supplementary Material S2). Homozygous observations for each gene are summarized together with carrier frequencies and prevalence estimates (Table 2).

**TABLE 2:** Carrier frequency and estimated prevalence of autosomal recessive diseases in Peru.

Gene	Disease	Heterozygous carriers (n)	Homozygous individuals (n’)	Observed carrier frequency	Estimated number of carriers in Peru^a^	Estimated disease prevalence (per 100,000 inhabitants)	Estimated number of affected individuals in Peru^a^
*GJB2*	Deafness, autosomal recessive 1A	7	0	0.0393	1350852	40.26	13,830
*ABCA4*	Cone-rod dystrophy 3/Stargardt disease 1/Retinitis pigmentosa 19	4	0	0.0225	771,916	12.92	4,437
*CNGA3*	Achromatopsia 2	4	0	0.0225	771,916	12.92	4,437
*DNAH17*	Spermatogenic failure 39	4	0	0.0225	771,916	12.92	4,437
*MUTYH*	Familial adenomatous polyposis 2	4	0	0.0225	771,916	12.92	4,437
*PAH*	Phenylketonuria	3	0	0.0169	578,937	7.22	2,481
*SRD5A2*	Pseudovaginal hypospadias perineoscrotal	3	0	0.0169	578,937	7.22	2,481
*BBS1*	Bardet-biedl syndrome	2	0	0.0112	385,958	3.19	1,097
*BTD*	Biotinidase deficiency	2	0	0.0112	385,958	3.19	1,097
*CFAP43*	Spermatogenic failure 3,919	2	0	0.0112	385,958	3.19	1,097
*DNHD1*	Spermatogenic failure 39	2	0	0.0112	385,958	3.19	1,097
*EVC*	Ellis van creveld syndrome	2	0	0.0112	385,958	3.19	1,097
*GAA*	Pompe disease, infantile-onset or late-onset	2	0	0.0112	385,958	3.19	1,097
*USH2A*	Retinitis pigmentosa 39/Usher syndrome type IIA	2	0	0.0112	385,958	3.19	1,097
*TNFRSF13B*	Common variable immunodeficiency 2/Immunoglobulin a 2 deficiency	2	0	0.0112	385,958	3.19	1,097
*LAMA2*	Muscular dystrophy, congenital, merosin deficient or partially deficient/Muscular dystrophy, limb-girdle, autosomal recessive 23	1	2	0.0056	192,979	19.73	6,776
*FANCG*	Fanconi anemia, complementation group G	1	1	0.0056	192,979	7.10	2,439
125 autosomal recessives disorders		1	0	0.0056	23,736,405	0.79	33,526

^a^ Estimated using Hardy–Weinberg equilibrium and national population estimates from the National Institute of Statistics and Informatics (INEI). These values represent exploratory theoretical projections derived from the study cohort and should not be interpreted as population-based estimates.

According to biological function or affected organ system, the most frequent variants were those related to inborn errors of metabolism, vision and retinal disorders, neurological and neurodevelopmental diseases, hearing disorders, and immunodeficiencies (Table 3).

**TABLE 3:** Categories of recessive diseases at INSN.

Category	n	%	Estimated prevalence of affected individuals per 10,000 inhabitants
Inborn errors of metabolism	34	20.0	114.4
Visual disorders/Retinal disorders	20	11.8	35.7
Neurological and neurodevelopmental disorders	20	11.8	35.7
Hearing loss/auditory disorders	12	7.1	12.2
Immunodeficiencies	12	7.1	12.2
Genodermatosis/skin and connective tissue disorders	10	5.9	8.4
Hematological diseases	9	5.3	6.7
Infertility	9	5.3	6.7
Ciliopathies and ciliopathy-associated multisystem syndromes	8	4.7	5.3
Endocrine disorders	8	4.7	5.3
Neuromuscular diseases	7	4.1	4.0
Cardiovascular diseases	4	2.4	1.3
Skeletal disorders	4	2.4	1.3
Cancer susceptibility disorders	4	2.4	1.3
Renal and cystic diseases	3	1.8	0.7
Hepatic diseases	2	1.2	0.3
Pulmonary diseases	2	1.2	0.3
Others	2	1.2	0.3

**FIGURE 2:**
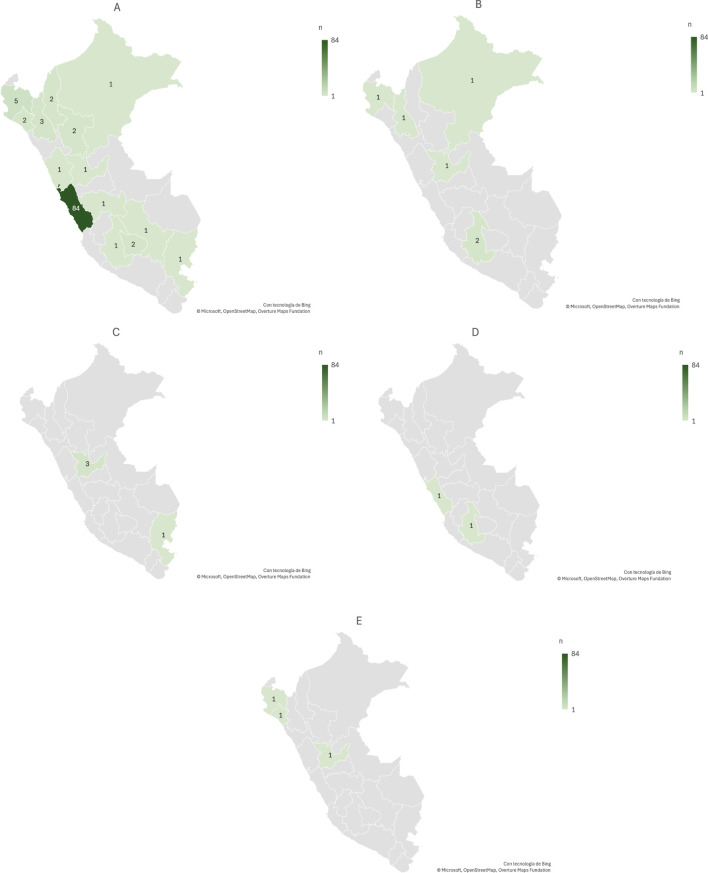
Geographic distribution of participants and frequency of selected genes according to the departmental origin of their grandparents in Peru. **(A)** Frequency distribution of carrier patients according to their residence. **(B)** *GJB2* variants: origin of maternal and paternal grandparents. **(C)** *CNGA3* variants: origin of maternal and paternal grandparents. **(D)** *DNAH17* variants: origin of maternal and paternal grandparents. **(E)** *MUTYH* variants: origin of maternal and paternal grandparents.

When evaluating frequency according to current place of residence, most patients were from Lima, followed by Piura and Cajamarca (Figure 2A). After selecting patients whose 4 grandparents originated from the same department, the most frequently identified genes were *GJB2*, *ABCA4*, *CNGA3*, *DNAH17*, *MUTYH*, and *PAH*. In relation to *GJB2*, associated with sensorineural hearing loss, the most frequent department of origin among grandparents was Ayacucho. In the case of *ABCA4*, associated with retinal diseases, all cases corresponded to individuals with grandparents originating from Lima. Likewise, variants in the genes *DNAH17* (male infertility), *MUTYH* (colorectal cancer susceptibility), and *PAH* (phenylketonuria) were observed in patients whose grandparents originated from the departments of Puno, Huánuco, Ayacucho, Piura, and Arequipa (Figures 2B–E). When evaluating whether any geographic region was associated with a higher carrier frequency, no significant differences were observed. No differences in prevalence ratios were observed according to patient origin or grandparent ancestry in relation to carrier frequency (Supplementary Material S3).

## 4 Discussion

The mean number of heterozygous pathogenic or likely pathogenic variants associated with autosomal recessive diseases was approximately one per individual, which is lower than that reported in European populations, where an average of at least two such variants per individual has been observed (Fridman et al., 2021). In some European subpopulations, such as Romania, up to 1.77 variants per individual have been reported (Gug et al., 2025). A Colombian study reported an average of 1.7 variants per individual (Idárraga et al., 2025). Nevertheless, our finding is relatively similar to that reported in Brazil, with a mean of 1.32 variants per individual (median = 1, SD = 1.188, maximum value of 5) (Quaio et al., 2021). The proportion of individuals without recessive variants was similar to that observed in Colombia, Brazil, and Romania (36.5% vs. 25%–29.5%) (Quaio et al., 2021; Gug et al., 2025; Idárraga et al., 2025), whereas a study from Northern India found that approximately 74% of individuals were non-carriers, suggesting a lower burden of recessive disease-associated variants in that population (Singh et al., 2020). Likewise, the proportion of individuals with three or more variants was comparable to that reported in Romania (7.3% vs. 7.5%) (Gug et al., 2025).

Carrier frequency associated with hearing loss caused by variants in *GJB2* was consistent with previous reports in Romania (0.0284 vs. 0.0393) (Riza et al., 2022). Similar frequencies have also been reported in Asian populations, including Japan (0.0373) and China (0.0286). Although the spectrum of pathogenic variants differed among populations, these findings suggest that pathogenic *GJB2* variants constitute a common cause of autosomal recessive hearing loss across diverse ethnic groups (Taniguchi et al., 2015; Liu et al., 2023). Regarding variants causing retinal disorders associated with *ABCA4*, the observed carrier frequency was lower than that reported in populations from Arabia and Europe (4.3%–6.4% vs. 2.3%) but similar to that observed in a Chinese population (2.44%) (Riveiro-Alvarez et al., 2009; Liu et al., 2024; Jain et al., 2025). Within inborn errors of metabolism, the carrier frequency of pathogenic variants in *PAH* was intermediate compared with frequencies reported in other populations, being higher than that reported in a Japanese cohort (0.00867) and lower than that observed in a population from the Karachay-Cherkess Republic in Russia (0.0625). Notably, the frequency observed in our cohort was similar to that reported in a Chinese population (0.0221) (Gundorova et al., 2018; Yamaguchi-Kabata et al., 2019; Liu et al., 2024).

When analyzing disease groups sharing a common organ system or pathophysiological mechanism, carriers of inborn errors of metabolism represented approximately one quarter of the cohort. In Brazil, this frequency reached 48.8% (Quaio et al., 2022), whereas a South Asian study reported a carrier frequency of approximately 5.5% (Bylstra et al., 2019). However, the estimated number of affected individuals per 10,000 inhabitants would be higher in our population (114 vs. 26.39) (Quaio et al., 2022).

Up to one third of the global population is estimated to be a carrier of a neuromuscular or retinal disorder. In our study population, the frequency reached 23.6% (Hanany et al., 2020; Choi et al., 2024). The frequency of affected individuals with retinal disease per 10,000 inhabitants was lower than previously reported (35.7 vs. 7.2) (Hanany et al., 2020). Similarly, the proportion of individuals affected by neuromuscular disorders was lower (3.57 vs. 24.3 per 10,000 individuals) (Choi et al., 2024).

Regarding primary immunodeficiencies of autosomal recessive inheritance, the estimated frequency of affected individuals (1.22 per 1,000) was higher than previously reported, where total primary immunodeficiency prevalence across all inheritance types ranges between one per 1,000 and one per 5,000 individuals (Schmitt and Cooper, 2021).

The estimated frequency of affected individuals with infertility-related recessive disorders was also elevated (6.7 per 10,000 individuals). Previous studies estimate prevalence ranging from one in 10,000 to one in 100,000 among women (Ruotsalainen et al., 2024).

Autosomal recessive sensorineural hearing loss presents highly variable frequencies globally (5–97 per 100,000 individuals). The proportion of affected individuals in our population was estimated at 122 per 100,000 individuals (Chakchouk et al., 2019).

The prevalence of individuals affected by autosomal recessive hematological disorders varies from fewer than one per million to more than one per 1,000 in endemic regions of hemoglobinopathies, with intermediate values for hereditary thrombotic thrombocytopenic purpura and familial hemophagocytic lymphohistiocytosis. Ethnic heterogeneity and consanguinity are determining factors in the frequency of these disorders (Hansen et al., 2020; Fan et al., 2023; Henter, 2025). In this context, the frequency observed in the present study was 6.7 per 10,000 individuals.

The variability in the carrier frequency of these disorders depends on several factors, such as internal and external migration patterns and genetic drift. The genetic landscape of Latin America is shaped by complex historical admixture among Native American, European, and African lineages, resulting in substantial population heterogeneity across countries and subregions. In Peru, this genetic architecture is predominantly characterized by a highly admixed background with variable ancestral proportions that differ regionally due to geographic, cultural, and historical factors (Ruiz-Linares et al., 2014; Borda et al., 2026).

In the literature, founder and bottleneck effects have been proposed as mechanisms that can contribute to differences in allele frequencies across populations. The founder effect occurs when a small proportion of individuals from a large population becomes isolated and forms a “new population” (Jain et al., 2021). In Peru, geographic isolation has historically driven founder effects in specific regions, such as the insular communities of *Taquile* and *Amantaní* in Lake Titicaca (Puno) or in historically isolated communities within the Piura region (Sandoval et al., 2004; Purizaca-Rosillo et al., 2017). The bottleneck effect occurs when a large population is drastically reduced due to environmental factors, leading to the non-random selection of certain variants, which may increase the population frequency of a genetic disease (Jain et al., 2021). In Latin America, large-scale demographic processes following European contact, together with subsequent admixture events, have contributed to the current genetic structure of populations. However, in the present study, these mechanisms are not directly assessed, and therefore these interpretations remain speculative in the absence of formal population genetic analyses. Alternatively, the observed variability in recessive variants may reflect baseline population-wide carrier frequencies or methodological differences across studies. This is supported by our finding that most detected variants are enriched in our cohort compared to global databases, aligning with the hypothesis that regional demographic history shapes the current genetic architecture of the Peruvian population.

Although all variants included in this study were analyzed as carrier findings in genes associated with autosomal recessive disorders, the clinical implications of heterozygosity are not necessarily equivalent across all genes. Some genes identified in our cohort, such as *MUTYH, SERPINA1*, and *TNFRSF13B*, have been associated with variable penetrance, increased disease susceptibility, or phenotypic effects in heterozygous individuals. Therefore, carrier status should be interpreted within the context of the specific gene and its known inheritance pattern, and direct comparisons across genes should be made with caution.

Among the limitations of this study, we were unable to determine whether the variants were founder mutations or *de novo*, as has been assessed in studies from Saudi Arabia (Abouelhoda et al., 2016). We did not include the frequency of copy number variants or multiple nucleotide variants, which may introduce bias for some diseases such as spinal muscular atrophy which is most commonly caused by deletion of exon 7 in *SMN1*, and infertility-hearing loss syndromes which may result from deletion of two genes (Cuellar et al., 2025). In addition, the dataset used in this study does not include genome-wide or ancestry-informative markers, and therefore analyses of population structure (e.g., principal component analysis or haplotype-based inference) could not be performed. Consequently, the geographic origin of participants was used only as a descriptive variable and should not be interpreted as a proxy for genetic ancestry or population stratification. An additional limitation relates to the sequencing platform (Ion Torrent), which has reduced sensitivity for detecting variants in homopolymeric regions and certain insertion/deletion (indel) events, particularly frameshift variants. Consequently, these variant types are underrepresented in our dataset. This is particularly relevant for cross-population comparisons, as most reference datasets are based on platforms with higher indel detection performance (e.g., Illumina-based sequencing). Therefore, differences in variant burden between populations should be interpreted cautiously, as they may reflect both true biological variation and platform-dependent detection bias. In addition, the sample size was relatively small. The cohort corresponds to a population undergoing molecular testing due to high suspicion of genetic disease, which does not imply population representativeness in a genetic epidemiological sense. Consequently, estimates derived from Hardy–Weinberg equilibrium should be interpreted with caution, as the study population does not constitute a random sample of the general population. Therefore, the estimated frequencies of carriers and affected individuals should be regarded as exploratory and theoretical rather than as direct population-based estimates for Peru and may overestimate the true prevalence of these conditions in the general population.

Despite the nationwide catchment area of the national referral center where testing was performed (INSN), geographic access barriers remain. Although the institution proportionally serves a large segment of the population compared to other healthcare systems, most participants originated from the coastal region. This partially reflects the demographic distribution of Peru, where the largest proportion of the population resides; however, significant constraints persist for sample delivery from the most isolated regions, compounded by the fact that many patients affected by genetic diseases cannot travel to Lima. Consequently, geographic comparisons may have limited statistical power and should be interpreted as exploratory rather than confirmatory. Finally, because the study included a relatively small sample, geographic and demographic factors were evaluated exclusively through unadjusted bivariate analyses. These regional comparisons are exploratory, lack statistical power to confirm complex demographic dependencies, and require validation in larger, population-based cohorts.

In this clinical cohort of pediatric patients undergoing exome sequencing at a national referral center in Peru, the mean number of heterozygous pathogenic variants associated with autosomal recessive diseases was close to one per individual, slightly lower than reported in European and Latin American populations. However, the proportion of individuals without recessive variants and the distribution of individuals with multiple variants were comparable to previous studies, suggesting that the overall burden of recessive variants in our population follows a similar international pattern.

Gene-specific analyses demonstrated heterogeneity in carrier frequencies across disorders. While frequencies of pathogenic variants in *GJB2* and *PAH* were generally comparable to those reported in other populations, *ABCA4*-associated retinal disorders showed lower carrier frequencies than those reported in European and Middle Eastern populations. Likewise, estimated prevalence of affected individuals across disease groups showed variation compared with previous studies, particularly in retinal disorders, neuromuscular diseases, primary immunodeficiencies, and autosomal recessive hematological disorders.

Overall, this study provides a description of the distribution of pathogenic and likely pathogenic heterozygous SNVs found by in a clinical exome sequencing cohort from a national referral center in Peru. These findings should be interpreted within the context of a clinically ascertained population and may serve as a reference for future population-based genomic studies in the region.

## Data Availability

The raw data supporting the conclusions of this article will be made available by the authors, without undue reservation.
